# A Case Report of Pipkin Type II Femoral Head Fracture Managed by Ganz Safe Surgical Dislocation and Headless Compression Screw (Herbert Screw) Fixation: A Stepwise Technical Description and Clinical Outcome

**DOI:** 10.7759/cureus.110765

**Published:** 2026-06-13

**Authors:** Sasidhar MVS, Prajwal Reddy, Venkata Vinay Atluri, K Uma Maheshwar

**Affiliations:** 1 Department of Orthopaedics, Kamineni Institute of Medical Sciences, Narketpally, IND

**Keywords:** femoral head fracture, ganz surgical hip dislocation, harris hip score, headless compression screw, herbert screw, medial femoral circumflex artery, pipkin type ii fracture, posterior hip dislocation, trochanteric flip osteotomy

## Abstract

Posterior hip dislocation with a concurrent shear fracture of the femoral head is an uncommon high-energy injury, and when the displaced fragment involves the weight-bearing dome cranial to the fovea capitis, anatomical reduction is essential to preserve joint kinematics and reduce the risk of late avascular necrosis and post-traumatic arthritis. We describe a 23-year-old male motorcyclist who sustained an isolated right posterior hip dislocation with a Pipkin Type II fragment of the superomedial femoral head. Closed reduction was achieved within four hours of injury (total time from injury to definitive reduction was 3 hours and 40 minutes, within the recognised six-hour vascular-protective window). Post-reduction computed tomography characterised a non-comminuted suprafoveal fragment measuring 22 × 18 mm, with an intact femoral neck and acetabulum. Definitive open reduction and internal fixation were performed via a Kocher-Langenbeck approach with Ganz trochanteric flip osteotomy. The fragment was anatomically reduced and secured with three Herbert headless compression screws - two of 4 mm × 18 mm placed antegrade from the articular surface of the fragment into the parent femoral head in a divergent configuration, and one of 4 mm × 24 mm placed retrograde from the parent head as a supplementary lag screw - all three screws crossing the fracture line. The trochanteric osteotomy was refixed with three 3.5 mm cortical screws. At 12 months postoperatively, the patient was pain-free, ambulating without aids, and had returned to his previous level of activity, with a Harris Hip Score of 98, a modified Merle d'Aubigné-Postel score of 17, a Tönnis grade of 0, and no radiographic evidence of avascular necrosis of the femoral head or heterotopic ossification. Surveillance is continuing in view of the recognised possibility of late-onset avascular necrosis presenting two to five years following injury. This report provides a stepwise technical description of the Ganz surgical hip dislocation combined with a divergent antegrade plus retrograde lag headless compression screw construct for isolated Pipkin Type II fixation, intended to complement existing series-level literature with reproducible operative detail.

## Introduction

Although traumatic posterior dislocation of the hip is itself an uncommon presentation in elective orthopaedic practice, an even smaller subgroup of these patients sustain a concurrent shear fracture of the femoral head, a combination identified in roughly 1 in 10 to 1 in 20 posterior dislocations in published cohorts [1]. The injury is overwhelmingly the consequence of high-energy vehicular trauma: with the hip flexed against a fixed surface such as a dashboard or motorcycle fuel tank, an axially transmitted load drives the femoral head against the posterior acetabular wall, shearing off a fragment as the head exits the socket [2]. Pipkin’s 1957 schema retains practical primacy in describing the resulting injury: a fragment lying caudal to the fovea capitis (type I) spares the loaded portion of the head, whereas a fragment lying cranial to the fovea (type II) carries away a portion of the weight-bearing dome; the presence of an additional femoral neck or acetabular wall fracture upgrades the injury to type III or type IV, respectively [3]. In the AO/OTA classification, the present injury corresponds to 31-C1.

The complications associated with operative management of Pipkin Type II fractures are well documented and form an essential part of preoperative counselling. The dominant long-term concern is post-traumatic avascular necrosis of the femoral head, the vascular supply of which depends almost entirely on the deep branch of the medial femoral circumflex artery (MFCA); reported avascular necrosis (AVN) rates following Pipkin Type II injuries treated by mixed traditional approaches range from 6% to 24%, with delay to reduction, the nature of the surgical approach, and the integrity of the MFCA at presentation all influencing risk [4]. Secondary post-traumatic osteoarthritis may follow even an anatomically reduced fragment if the loaded articular surface is incongruent by a millimetre or more, and may require arthroplasty in the medium to long term [4]. Heterotopic ossification is encountered particularly with anterior (Smith-Petersen) approaches, with rates reaching 30% in some series [5]. Other recognised complications include surgical site infection, deep vein thrombosis and pulmonary embolism, sciatic nerve injury (most commonly traction neuropraxia), implant failure or backout, non-union of the trochanteric osteotomy where this approach is used, and recurrent dislocation. The favourable functional benefit of timely operative fixation - restoration of articular congruity, preservation of joint kinematics, and reduction in the risk of secondary arthrosis - must therefore be weighed against this specific complication profile, and the surveillance window for late AVN extends to at least five years following injury.

The clinical problem posed by a type II fragment is distinct from that of a type I fragment. Because the fracture surface forms part of the loaded articulation, even a one- or two-millimetre step renders the joint kinematically abnormal at the very arc of motion through which it is loaded and predisposes the cartilage to early degenerative change [4]. Two traditional exposures have been used to address this. An anterior Smith-Petersen window delivers the offending fragment directly into the wound but at the cost of opening the anterior capsule and stripping muscle from the iliac wing, with a recognised tendency to seed heterotopic bone [5]. A posterior Kocher-Langenbeck window protects the dominant vascular pedicle to the head but provides only a tangential view of an anterosuperior fragment; the head must be levered or sometimes briefly subluxed to gain access, manoeuvres that introduce their own ischaemic risk to a head already devascularised by the original dislocation [6].

A third exposure, introduced in 2001 by Ganz and colleagues from Bern, was designed expressly to resolve this dilemma between exposure and vascular safety. By flipping the greater trochanter as a digastric bone block carrying the gluteus medius proximally and vastus lateralis distally, and then opening the capsule along a Z-shaped path that protects the posterosuperior retinacular vessels, the femoral head can be lifted out of the acetabulum and rotated under direct vision while its principal vascular pedicle, the deep branch of the medial femoral circumflex artery, remains undisturbed in the soft tissue between piriformis and obturator externus [7,8]. The exposure was originally conceived for femoroacetabular impingement surgery, but the same circumferential access has since proved useful for complex acetabular fractures, slipped epiphyses, and selected femoral head fractures, including improved outcomes in Pipkin type IV injuries [9,10]. Application of the technique specifically to isolated Pipkin type II injuries, however, remains thinly described in the indexed literature.

Despite this growing evidence base, published reports remain dominated by series-level summaries rather than detailed technical descriptions. A PubMed search performed on 15 May 2026 using the terms “Pipkin” AND “Ganz surgical hip dislocation” between 2001 and 2025 retrieved 23 indexed publications, the majority of which are retrospective series that document outcomes without providing reproducible step-by-step intraoperative detail. There is, in particular, a paucity of granular technical reports from South Asian centres, where the Smith-Petersen and Kocher-Langenbeck approaches predominate in routine practice. We therefore present a single case of isolated Pipkin type II fracture managed by Ganz surgical hip dislocation, with three explicit aims: (i) to provide a stepwise, reproducible technical narrative of the osteotomy, capsulotomy, surgical dislocation, fragment reduction, and headless compression screw fixation; (ii) to report objective functional and radiographic outcomes using standardised scoring instruments (Harris Hip Score, modified Merle d’Aubigné-Postel score, Brooker classification, and Tönnis grade); and (iii) to position the present case against the cumulative published experience through a comparative summary of contemporary series. This case report was prepared in accordance with the CARE guidelines.

## Case presentation

This is a single-patient diagnostic and therapeutic case report describing the operative management and postoperative outcome of a Pipkin Type II femoral head fracture, prepared in accordance with the CARE (Case Report) guidelines.

A 23-year-old male student of South Asian (Indian) ethnicity was brought to the casualty department within 90 minutes of a road traffic accident in which he was a motorcycle rider involved in a head-on collision. He presented with severe right hip pain and inability to bear weight. There was no loss of consciousness, vomiting, or seizure activity, and no clinical or radiological evidence of head injury. He had no comorbidities, no previous surgical history, and was not on any medications. Written informed consent was obtained from the patient for publication of this case report and any accompanying clinical images. Institutional ethics committee approval was obtained for publication.

General and local examination

Primary and secondary surveys were conducted in accordance with the Advanced Trauma Life Support (ATLS) protocol. On primary survey, the patient was haemodynamically stable. Vital parameters were as follows: pulse 92 beats per minute, blood pressure 122/78 mm Hg, respiratory rate 18 per minute, oxygen saturation 99% on room air, and Glasgow Coma Scale (GCS) 15/15. Cardiovascular, respiratory, abdominal, and neurological systemic examinations were within normal limits. The cervical spine was clinically clear. The secondary survey did not reveal any other long-bone or visceral injury.

On local examination of the right hip, the limb was held in the classical attitude of posterior hip dislocation: flexion at the hip, adduction, internal rotation, and apparent shortening. Superficial abrasions were noted over the right knee, but no open wound was present around the hip. There was no swelling or ecchymosis over the greater trochanteric region. On palpation, local tenderness was elicited over the gluteal region. The right greater trochanter was felt to be elevated and posteriorly displaced relative to the contralateral side. No bony crepitus was appreciated. All active and passive movements of the right hip were severely restricted and painful; any attempt to extend or externally rotate the hip elicited spasms and severe pain. True shortening of approximately 2 cm was noted on the right side (anterior superior iliac spine to medial malleolus), with apparent shortening of similar magnitude.

Distal neurovascular status was carefully documented and was intact. Sensations were preserved over the lateral leg, dorsum of the foot, sole, and lateral aspect of the foot. Motor power was 5 of 5 in the tibialis anterior, extensor hallucis longus, peroneal group, gastrocnemius, and intrinsic foot muscles. Dorsalis pedis and posterior tibial pulses were well felt and equal bilaterally. Capillary refill was less than two seconds. There was no clinical evidence of sciatic or femoral nerve injury. Examination of adjacent joints (right knee and ankle) and of the contralateral lower limb was unremarkable. There was no clinical evidence of ipsilateral posterior cruciate ligament injury or patellar fracture, which can accompany this mechanism.

Initial radiological evaluation and closed reduction

**Figure 1:**
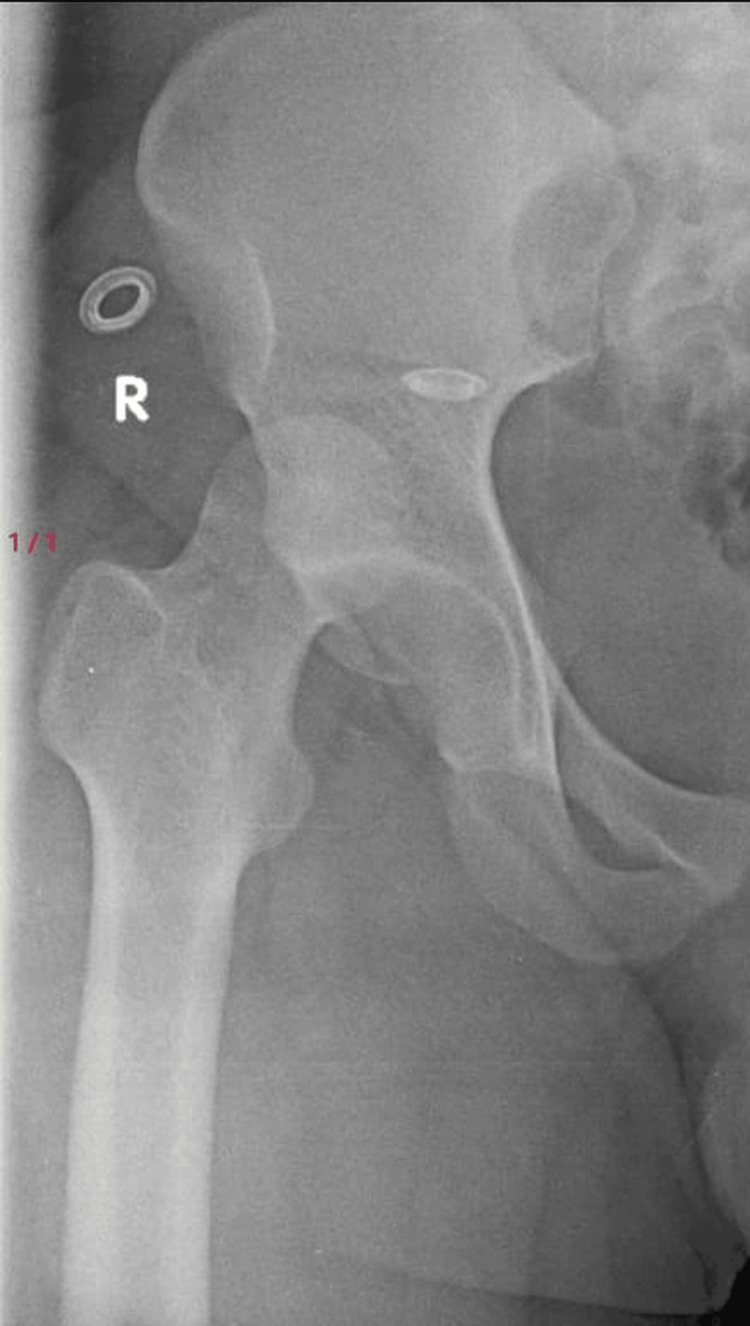
Pre-reduction anteroposterior radiographic view of the right hip on initial presentation. The radiograph demonstrates posterior dislocation, with the femoral head displaced superolaterally, lying outside the acetabular contour (orientation marker R denotes right side). Shenton’s line is disrupted on the right. There is no femoral neck or acetabular fracture appreciable on this initial screening view.

Plain radiographs of the pelvis with both hips in anteroposterior view confirmed posterior dislocation of the right hip, with the femoral head displaced superolaterally to the acetabulum (Figure 1). Shenton’s line was disrupted on the right side. The femoral neck and acetabulum appeared intact on initial screening. In view of the established time-sensitive nature of hip dislocation, with a recommended window of six hours to minimise the risk of avascular necrosis [11], the patient was prepared for emergent closed reduction.

Initial assessment included plain radiographs of the pelvis and right hip, which demonstrated a posterior dislocation of the right hip with an associated femoral head fragment. No CT was obtained prior to reduction; the pre-reduction assessment was based on plain radiographs only. Closed reduction of the dislocation was performed under sedation in the emergency department within six hours of injury. Post-reduction plain radiographs confirmed concentric reduction of the hip joint with no significant joint-space widening and identified the residual suprafoveal fragment, prompting cross-sectional imaging. Total time from injury to definitive reduction was 3 hours and 40 minutes, within the six-hour vascular-protective window.

Post-reduction imaging and definitive diagnosis

**Figure 2:**
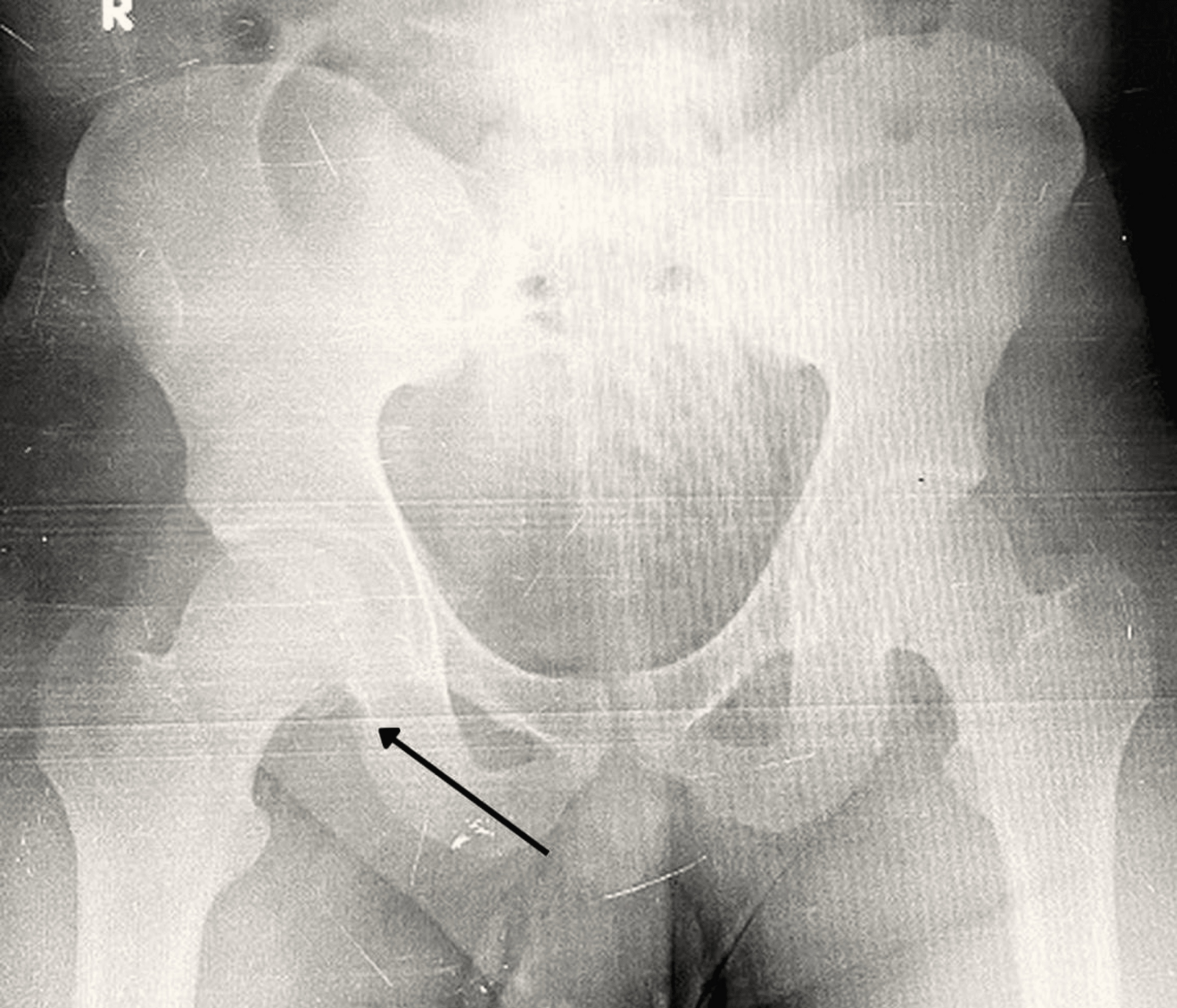
Post closed reduction anteroposterior radiograph of the pelvis with both hips. The arrow indicates the fracture site at the right femoral head, demonstrating concentric reduction of the femoral head within the acetabulum, with the Pipkin type II fragment located cranial to the fovea capitis. The femoral neck and acetabulum seem intact.

Repeat anteroposterior pelvis radiographs confirmed concentric reduction of the femoral head; however, they also revealed a fracture fragment of the femoral head located cranial (superior) to the fovea capitis, consistent with a Pipkin Type II injury pattern (Figure 2). Following confirmed concentric reduction, a non-contrast computed tomography (CT) scan of the right hip was performed with thin-cut axial sections and coronal and sagittal reconstructions to characterise the fracture pattern definitively, exclude an occult femoral neck or acetabular component, and guide operative planning. CT confirmed a single well-defined suprafoveal fracture of the femoral head consistent with Pipkin Type II morphology: a non-comminuted fragment measuring 22 × 18 mm located in the superomedial weight-bearing portion of the head and cranial to the fovea capitis, with concentric reduction of the femoral head within the acetabulum, no intra-articular loose body, an intact femoral neck (excluding Pipkin Type III), an intact posterior wall and rim of the acetabulum (excluding Pipkin Type IV), and no evidence of marginal impaction. Joint congruity after reduction was satisfactory. The CT findings directly informed the operative strategy: the fragment size and suprafoveal location were judged amenable to anatomical reduction and internal fixation rather than excision, and the Ganz surgical hip dislocation approach was selected to permit safe circumferential access to the fracture site while preserving the deep branch of the medial femoral circumflex artery. The archived CT images were of insufficient resolution for publication-grade reproduction and have therefore not been included as a figure; the textual description above provides the complete CT findings.

Pre-operative planning

Routine pre-operative blood investigations, including complete blood count, coagulation profile, renal and liver function tests, electrolytes, blood grouping and cross-matching, viral markers, electrocardiogram, and chest radiograph, were within normal limits. The patient was assessed as American Society of Anesthesiologists (ASA) Physical Status Grade I preoperatively, indicating a normal, healthy patient with no systemic disease. The patient and his family were counselled in detail regarding the nature of the injury; the rationale for surgical fixation (involvement of the weight-bearing portion mandating anatomical reduction); the planned operative approach, including the trochanteric osteotomy; the risk of avascular necrosis (reported between 6% and 24% in mixed-approach historical series [4,11]); heterotopic ossification; post-traumatic osteoarthritis; infection; neurovascular injury; non-union of the osteotomy; and the prolonged restricted weight-bearing protocol. Surgery was scheduled within 48 hours of injury, in keeping with the recommended window for definitive fixation of femoral head fractures [12,13].

Operative procedure

The patient was taken up under combined spinal-epidural anaesthesia. A bupivacaine spinal block provided intraoperative anaesthesia, while an epidural catheter was secured for postoperative analgesia. He was positioned in the left lateral decubitus position on a standard radiolucent operating table, with the right (operative) limb superior and prepared free to allow intraoperative manipulation. A pelvic positioner stabilised the pelvis perpendicular to the floor. All bony prominences were padded, and an axillary roll was placed under the dependent (left) axilla. The operative field, including the flank, gluteal region, hip, and the limb up to the knee, was painted and draped to allow free manipulation throughout the procedure. Prophylactic intravenous cefuroxime 1.5 g was administered 30 minutes prior to incision.

A modified Kocher-Langenbeck skin incision was made, centred over the greater trochanter and extending proximally in line with the fibres of the gluteus maximus and distally along the femoral shaft. Subcutaneous tissue and the fascia lata were incised in line with the skin incision. The fibres of the gluteus maximus were split bluntly along their orientation. The deep gluteal space was identified, and stay sutures were placed on the posterior border of the gluteus medius to retract it anteriorly. The trochanteric bursa was incised to expose the long external rotators. Particular care was taken to identify and protect both the piriformis tendon and the obturator externus throughout the procedure. The vascular rationale for this is specific: anatomical dissections by Gautier and colleagues have shown that the terminal feeders to the femoral head travel as the deep branch of the medial femoral circumflex artery, hugging the lower margin of the obturator externus before turning superiorly to enter the joint capsule near the piriformis insertion [8]. Any blade or retractor passed deep to the obturator externus is therefore positioned directly on the vessel that the entire operation is designed to preserve.

A digastric osteotomy of the greater trochanter was performed, such that the gluteus medius and minimus remained attached to the proximal trochanteric fragment, and the vastus lateralis remained attached distally, together forming the digastric sling. The osteotomy line was marked from the posterosuperior aspect of the greater trochanter, just lateral to the insertion of the piriformis tendon (preserving its attachment to the stable trochanter), running distally to exit lateral to the vastus ridge, producing a fragment approximately 1.5 cm thick. The osteotomy was completed using an oscillating saw under direct vision, with strict attention to keep the saw cuts lateral to the posterior border of the greater trochanter to protect the deep branch of the medial femoral circumflex artery. The mobile osteotomy fragment was reflected anteriorly along with its attached gluteus medius, gluteus minimus, and vastus lateralis.

**Figure 3:**
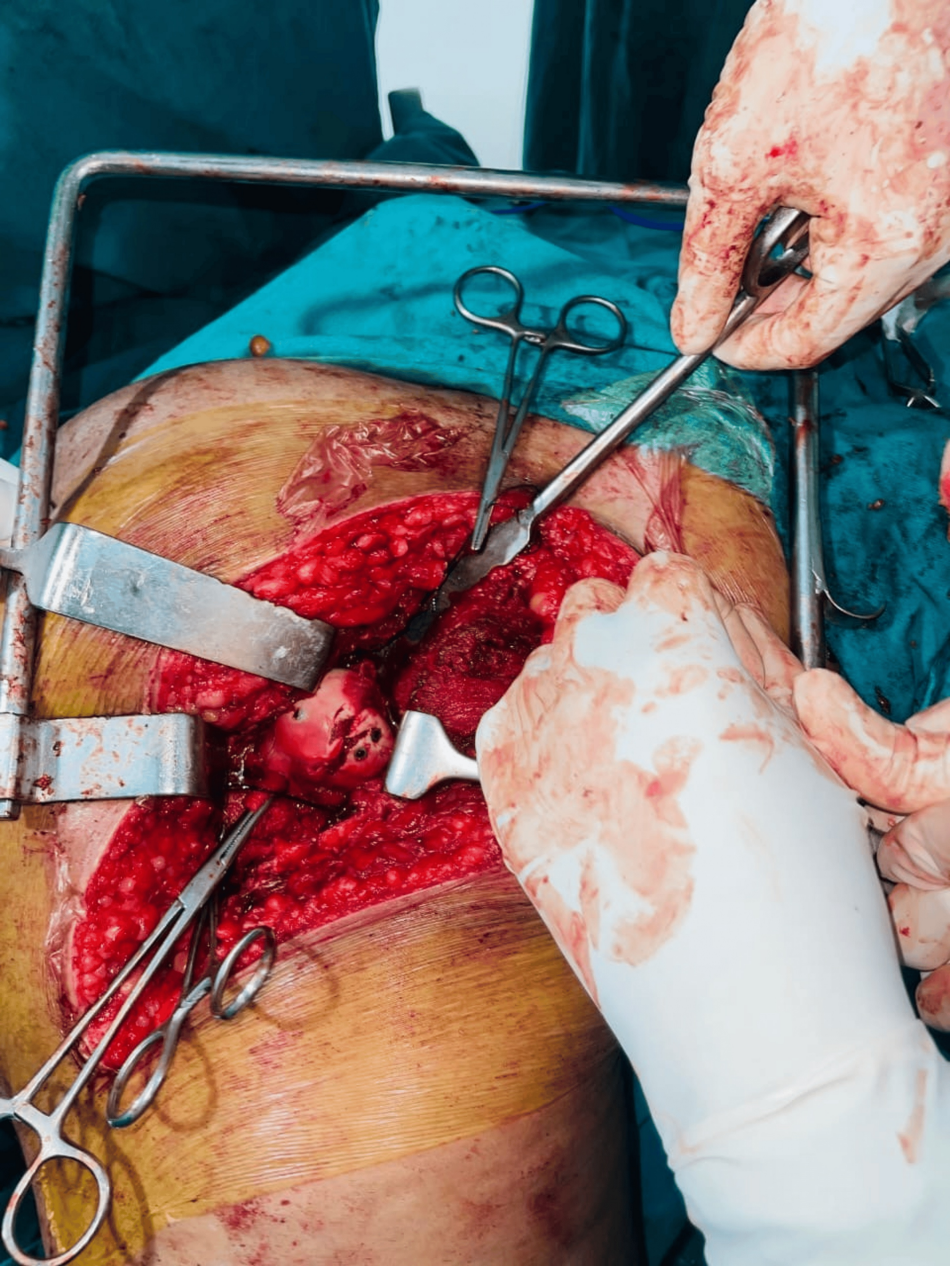
Intraoperative photograph showing the right hip exposed via the Ganz surgical hip dislocation approach. The patient is in the left lateral decubitus position. The digastric trochanteric flip osteotomy has been performed, and the trochanteric fragment (with attached gluteus medius, gluteus minimus, and vastus lateralis) has been reflected anteriorly. Through the Z-shaped capsulotomy, the femoral head is visible in the depth of the operative field, allowing complete 360-degree visualisation. A Charnley retractor frame stabilises the surgical exposure.

With the trochanteric fragment reflected, the underlying hip joint capsule was exposed and incised in three connected segments, forming the “Z” pattern described by Ganz. The central limb of the Z ran along the long axis of the femoral neck, the proximal limb turned anteriorly to follow the acetabular labrum just short of the piriformis insertion (the deliberate restraint at this turn protects the retinacular vessels entering the capsule from above), and the distal limb extended along the intertrochanteric ridge towards the vastus tubercle. With the capsule opened in this fashion, the hip was dislocated anteriorly under controlled conditions: flexion, external rotation, and adduction were applied with the leg dropped over the front of the table while the assistant applied gentle longitudinal traction. The femoral head delivered itself into the wound and could be rotated in any plane to inspect both articular surfaces in their entirety. The ligamentum teres was divided to permit the head to be lifted free for unobstructed inspection (Figure 3).

Direct visualisation confirmed a single Pipkin type II fragment originating from the superomedial weight-bearing surface, cranial to the fovea capitis. The fracture surfaces were gently irrigated with saline, and small clots and fibrinous debris were removed without disturbing the cartilage or subchondral bone. The fragment and its bony bed were inspected and found to be in excellent condition. No associated labral or chondral acetabular injuries were identified on direct inspection, an advantage of the 360-degree visualisation afforded by this approach [12]. The fragment was anatomically reduced onto the parent femoral head under direct vision, and reduction quality was assessed both visually and by palpation of the articular surface for any step-off. Anatomical congruity was achieved with no step or gap.

**Figure 4:**
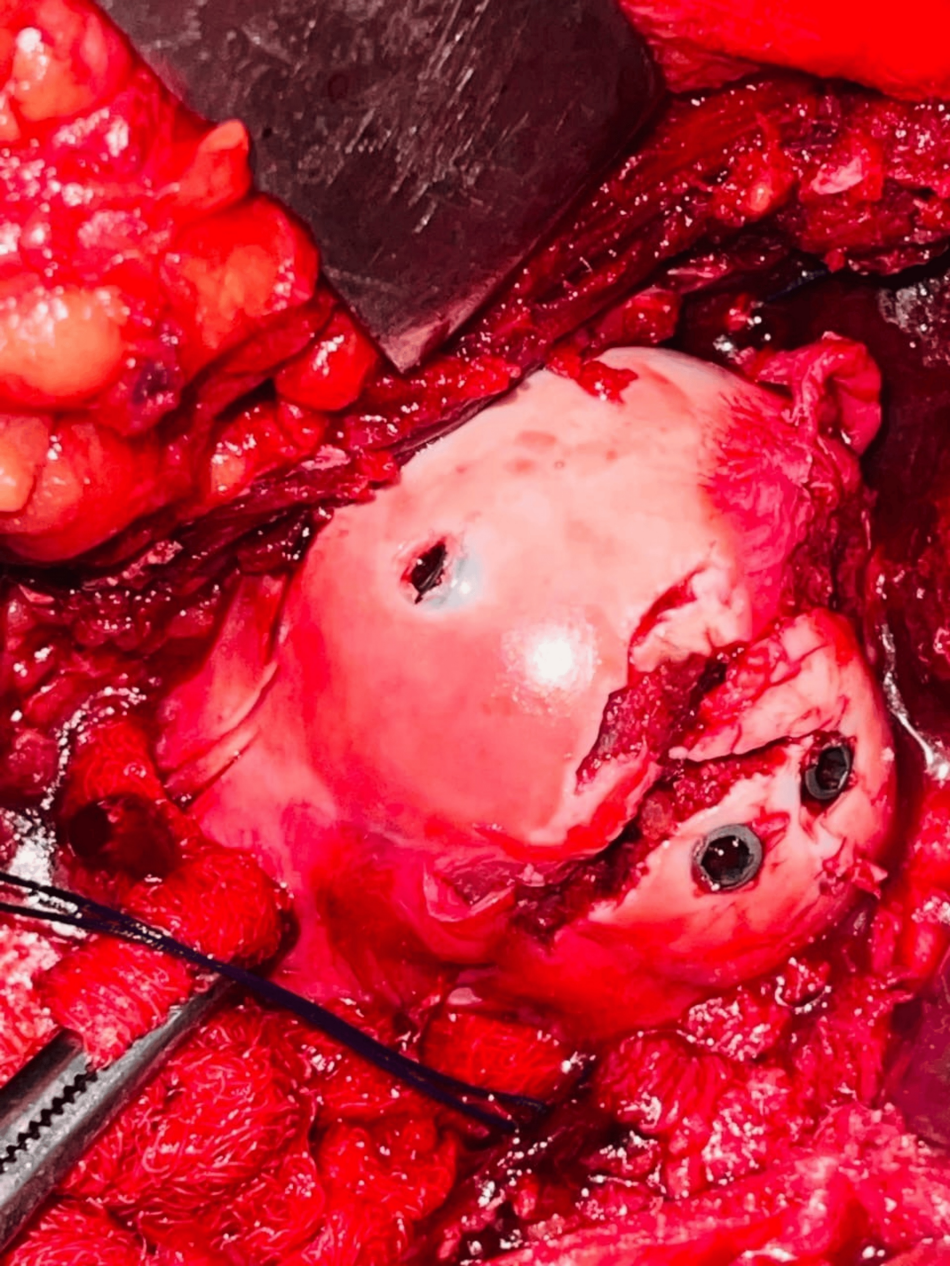
Intraoperative close-up photograph of the surgically dislocated femoral head following anatomical reduction and definitive fixation of the Pipkin type II fragment. Intraoperative close-up photograph of the surgically dislocated femoral head following anatomical reduction and definitive fixation of the Pipkin Type II fragment. The fracture line is clearly visible, separating the fragment (lower right) from the parent femoral head. Three Herbert headless compression screws have been used to secure the construct, all three crossing the fracture line: two screws of 4 mm × 18 mm placed antegrade from the articular surface of the fragment into the parent femoral head in a divergent configuration (heads visible on the fragment, countersunk beneath the cartilage), and one screw of 4 mm × 24 mm placed retrograde from the parent head as a supplementary lag screw (head visible on the parent head, also countersunk). The articular cartilage of the femoral head is preserved and intact.

Reduction was held provisionally with a 1.2 mm Kirschner wire passed perpendicular to the fracture plane. Stable definitive fixation was then constructed using three Herbert headless compression screws, selected to provide interfragmentary compression while permitting all screw heads to be buried beneath the articular cartilage - a critical requirement to avoid chondral damage to the acetabulum on subsequent reduction of the hip. Two screws of 4 mm × 18 mm were first inserted antegrade from the articular surface of the fragment into the parent femoral head in a divergent configuration; pilot holes were drilled perpendicular to the fracture plane and at divergent trajectories to provide rotational stability, depth was carefully measured, and both screws were seated such that the trailing thread was completely countersunk below the level of the surrounding articular cartilage. A third screw of 4 mm × 24 mm was then inserted retrograde from the lateral cortex of the parent femoral head, crossing the fracture line as a supplementary lag screw to provide an additional compression vector and enhance rotational stability of the construct; this screw was similarly countersunk at its entry point on the parent head. All three screws crossed the fracture line. Compression across the fracture was confirmed, the provisional Kirschner wire was removed, and the reduction was rechecked, demonstrating an anatomical, stable reduction with no chondral prominence (Figure 4). Screw position was further confirmed under intraoperative fluoroscopy in multiple planes, verifying appropriate length, divergent trajectory of the antegrade screws, complete subchondral seating of all three screw heads, and absence of intra-articular protrusion at any entry or exit point. Copious joint lavage was performed.

**Figure 5:**
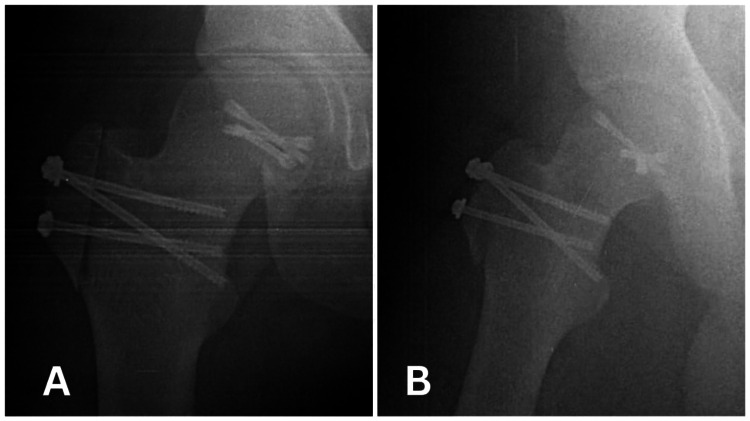
Initial post-operative day 0 radiograph of operated right hip. (A) Anteroposterior view. (B) Oblique view. Both views demonstrate (i) anatomical reduction of the femoral head fragment held by three 4 mm Herbert headless compression screws — two of 4 mm × 18 mm placed antegrade through the fragment, and one of 4 mm × 24 mm placed retrograde from the parent femoral head as a supplementary lag screw — all three crossing the fracture plane and countersunk beneath the articular cartilage; and (ii) anatomical reduction of the greater trochanteric flip osteotomy held by three definitive 3.5 mm cortical screws after replacement of the provisional Kirschner wires.

The hip was reduced into the acetabulum by reversing the dislocation manoeuvre (gentle traction with internal rotation and extension). Stability and range of motion were re-checked through full flexion, extension, abduction, adduction, and internal-external rotation; the hip remained concentric and stable throughout the entire arc. The capsule was repaired anatomically with absorbable sutures, restoring the Z-capsulotomy. The greater trochanteric fragment was reduced anatomically to its bony bed and provisionally held with Kirschner wires. It was then stably fixed with three 3.5 mm cortical screws in lag fashion. Compression and stability of the osteotomy were confirmed. The fascia lata was closed with a continuous absorbable suture, followed by subcutaneous tissue and skin closure in layers. A sterile dressing was applied. Estimated blood loss was approximately 350 mL, and total operative time was approximately 150 minutes. Final intraoperative fluoroscopy confirmed satisfactory fixation in two planes (Figure 5).

Post-operative course, rehabilitation, and follow-up

The patient was shifted to the post-anaesthesia care unit and subsequently to the male orthopaedic ward. Epidural analgesia was continued for 48 hours. Intravenous antibiotic prophylaxis was administered for 24 hours. Mechanical thromboprophylaxis with intermittent pneumatic compression and pharmacological prophylaxis with enoxaparin 40 mg subcutaneously once daily were initiated as per institutional protocol. Sutures were removed on post-operative day 12. The wound healed primarily without infection or dehiscence.

A graded weight-bearing protocol was followed. Strict non-weight-bearing on the right lower limb was maintained from week 0 to week 4; bed mobility and gentle isometric quadriceps and gluteal exercises were encouraged from day 1; and active assisted range-of-motion exercises within a pain-free arc were begun under physiotherapy supervision from day 2, avoiding extreme abduction or rotation that could stress the trochanteric osteotomy. From week 4 to week 8, partial weight-bearing was permitted with axillary crutches, progressing gradually as tolerated, with closed kinetic chain strengthening added. From week 8 onward, the patient progressed to full weight-bearing, confirmed by serial radiographs at the 4-week and 12-week visits showing union of the trochanteric osteotomy and incorporation of the femoral head fragment.

**Figure 6:**
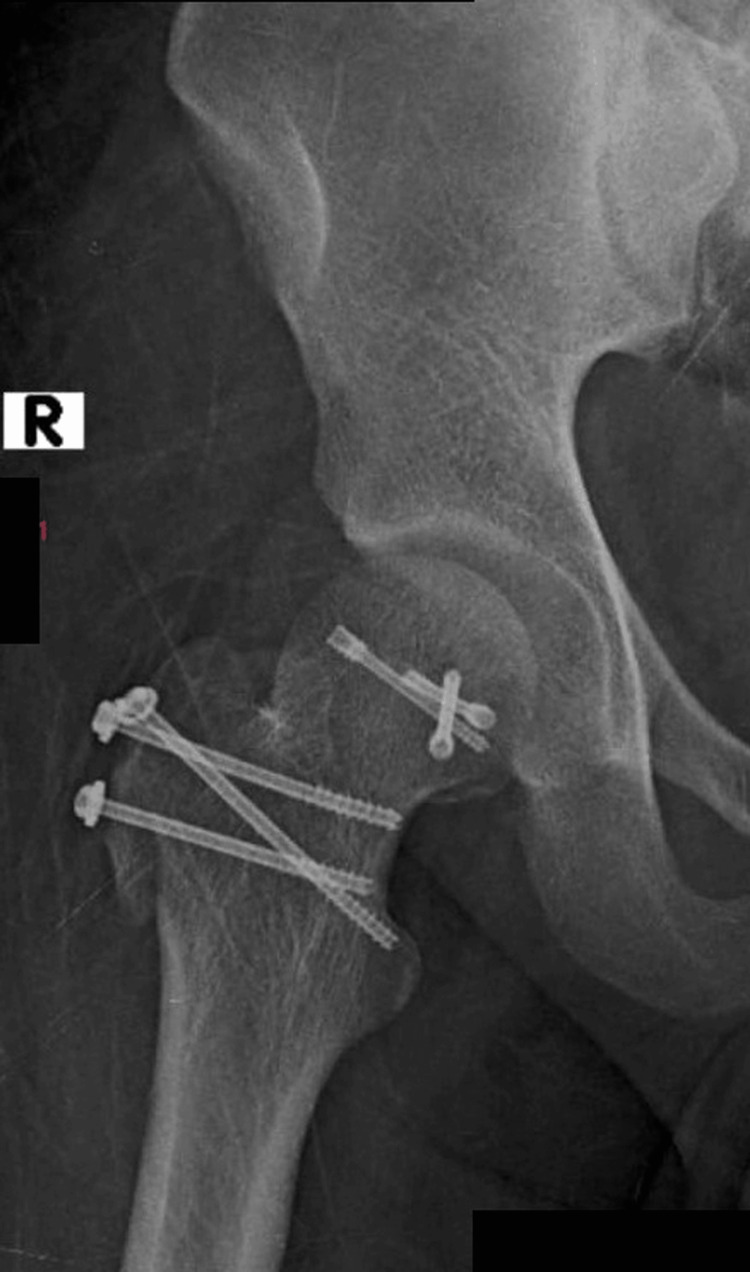
Four-week post-operative follow-up anteroposterior radiograph of the right hip. The radiograph demonstrates well-maintained anatomical reduction of the femoral head fragment with three countersunk headless compression screws, anatomical position of the greater trochanteric flip osteotomy held by three 3.5 mm cortical screws, a concentric hip joint, preserved joint space, no femoral head collapse, and no heterotopic ossification.

**Figure 7:**
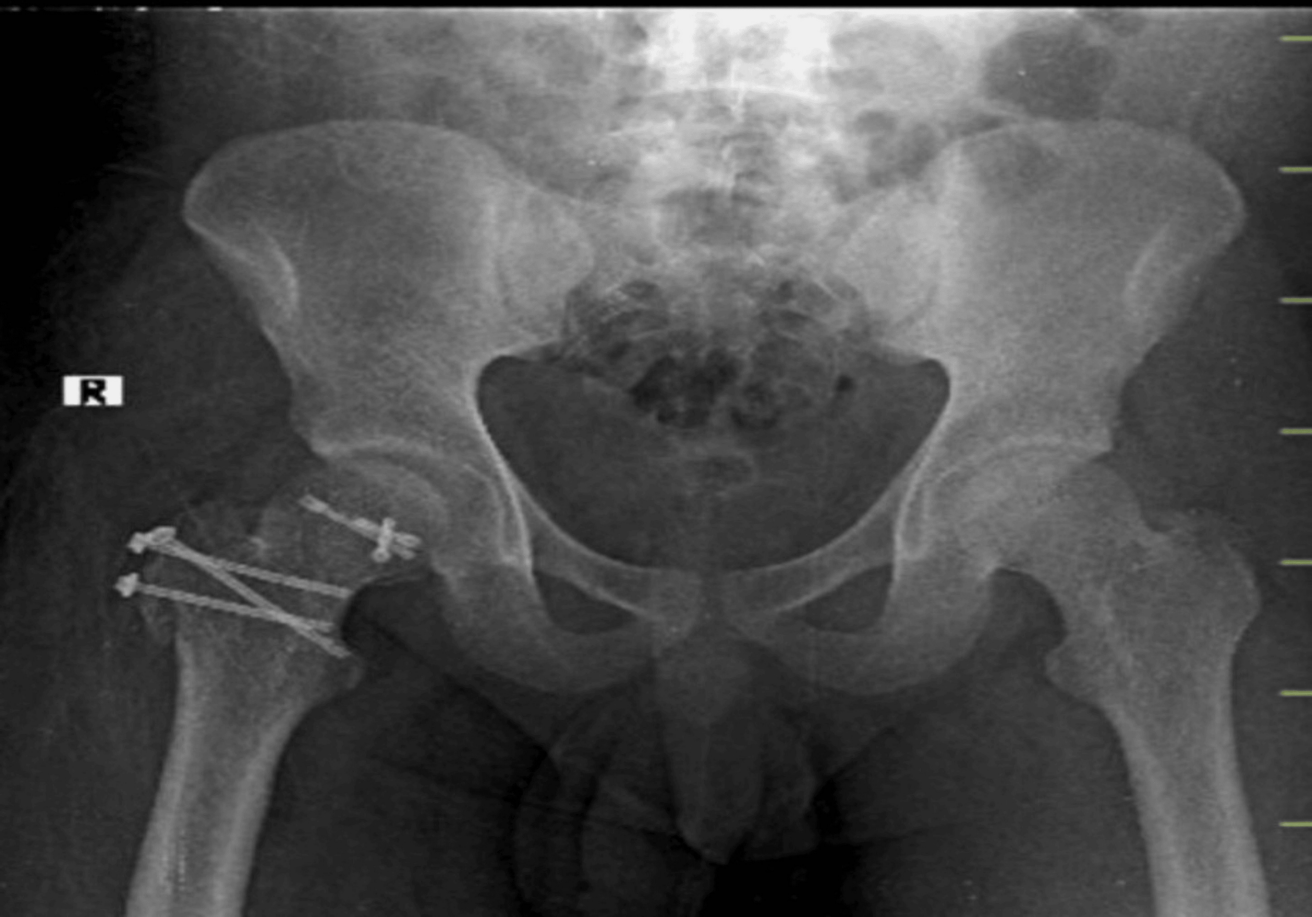
Four-week post-operative follow up anteroposterior radiograph of the pelvis with both hips. Four-week post-operative anteroposterior radiograph of the pelvis with both hips, demonstrating a well-maintained, concentric right femoral head with no screw penetration of the articular surface or joint space, and a symmetrical contralateral hip for comparison.

At the 4-week follow-up visit, radiographs demonstrated maintained anatomical reduction of the femoral head fragment, satisfactory position of the countersunk headless compression screws, anatomical position of the trochanteric flip osteotomy held by the cortical lag screws, a preserved joint space, and no signs of femoral head collapse (Figures 6, 7). The wound was healed, and the patient was clinically pain-free at rest. The active range of hip motion within a pain-free arc was satisfactory, and the patient was transitioned to partial weight-bearing with axillary crutches as per the rehabilitation protocol.

Structured clinical and radiographic follow-up continued at 4 weeks, 3 months, 6 months, and 12 months. Functional outcome was assessed using the Harris Hip Score and the modified Merle d’Aubigné-Postel score. Heterotopic ossification (HO) was assessed using the Brooker classification, and joint space was assessed using the Tönnis grade. By three months, the patient had achieved full weight-bearing without aids, had a full range of hip motion symmetrical to the contralateral side, and had returned to all activities of daily living. He reported no pain, no limp, and no functional limitation. The Harris Hip Score at three months was 96 of 100, the modified Merle d’Aubigné-Postel score was 17 of 18, the Brooker grade was 0, and the Tönnis grade was 0. Serial outcome measurements are summarised in Table 1.

**Table 1:** Serial functional and radiographic outcome measurements during 12-month follow-up. No HO (heterotopic ossification) and Tönnis grade 0 indicate no radiographic signs of osteoarthritis. *At 4 weeks, the patient was still in the protected non-weight-bearing phase, and functional scoring was deferred to the 3-month visit when weight-bearing had progressed.

Time point	Harris Hip Score (/100)	Merle d’Aubigné-Postel (/18)	Brooker grade (HO)	Tönnis grade
4 weeks	Not assessed*	Not assessed*	0	0
3 months	96	17	0	0
6 months	97	17	0	0
12 months	98	17	0	0

**Figure 8:**
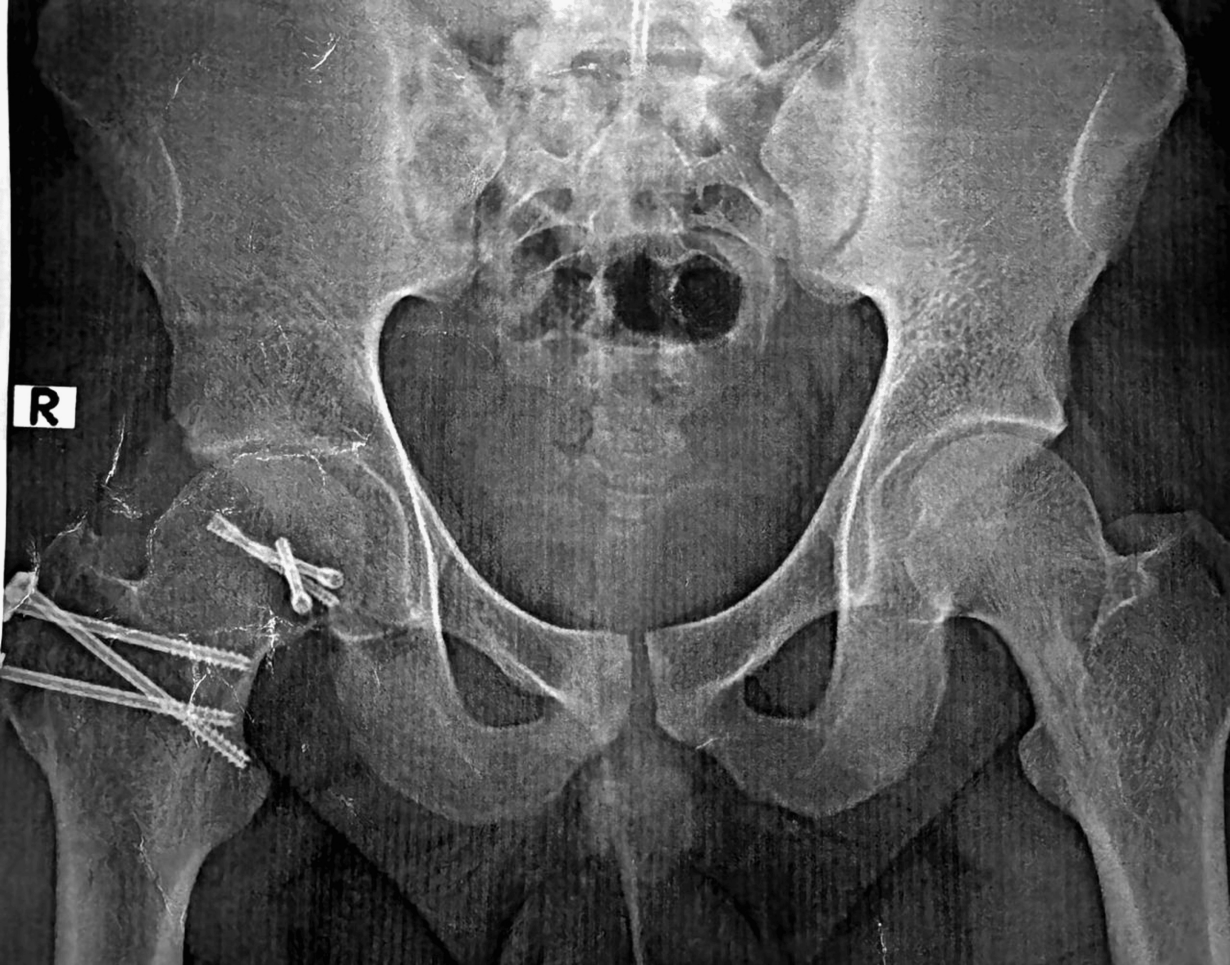
12 months post-operative anteroposterior radiograph of the pelvis with both hips. The radiograph demonstrates complete union of the greater trochanteric osteotomy on the operated right side, maintained reduction of the femoral head fragment with the countersunk headless compression screws in stable position, a concentric and symmetric right hip joint with a preserved joint space, no radiographic evidence of avascular necrosis of the femoral head, joint space narrowing or heterotopic ossification.

At 12 months, the patient was completely pain-free, ambulating without aids, fully returned to his student responsibilities and recreational activities, and reported subjective hip function indistinguishable from his uninjured side. The Harris Hip Score was 98 of 100, the modified Merle d’Aubigné-Postel score was 17 of 18, the Brooker grade was 0, and the Tönnis grade was 0. Radiographs at 12 months demonstrated maintained anatomical reduction of the femoral head fragment, complete union of the trochanteric osteotomy, no femoral head collapse, no joint space narrowing, no heterotopic ossification, and no implant-related complications (Figure 8). The patient remains under continued surveillance to monitor for late-onset avascular necrosis, which can present at 2 to 5 years post-injury [14].

Patient perspective

At the time of the injury, I was in severe pain and unable to bear weight on my right leg. I was reassured by the surgical team's clear explanation of the diagnosis and of the proposed surgical plan, particularly the rationale for choosing a specialised approach that protects the blood supply to the hip joint and reduces the risk of later complications. The early postoperative period required patience and disciplined adherence to the rehabilitation protocol, including a period of restricted weight-bearing and structured physiotherapy. With the support of the orthopaedic team, my recovery progressed steadily. More than one year after surgery, I have returned to my previous level of daily activity - walking, climbing stairs, and resuming my usual student responsibilities and recreational activities - without pain or limitation. I appreciate the careful explanation of the long-term surveillance plan, which has helped me understand the importance of ongoing follow-up. Overall, I am satisfied with the outcome of the surgery and with the quality of care I received.

## Discussion

Two competing surgical principles have historically shaped the management of Pipkin type II injuries: the desire for unobstructed visualisation of the fracture surface and the obligation to safeguard a vascular pedicle that has already been stressed by the dislocation event itself. The Smith-Petersen window satisfies the first principle handsomely - the anterosuperior fragment lies directly beneath the surgeon’s hand - but the broad capsular and muscular exposure required is the same anatomical insult that has been linked in published case series to a higher incidence of heterotopic bone formation, and the disruption of any residual anterior capsular contributions to head perfusion is an unwelcome additional burden on an already-injured head [5]. The Kocher-Langenbeck window inverts the trade-off: the vascular pedicle is left undisturbed, but the surgeon faces the fragment from behind, often through a narrow keyhole, and is tempted into the very head manipulation that the choice of approach was meant to avoid [6,13]. The Ganz dislocation, in its own way, refuses this trade-off altogether. By moving the femoral head out of the acetabulum under direct vision while keeping the deep branch of the medial femoral circumflex artery undisturbed in soft tissue, it offers what neither traditional approach can: full circumferential access without the cost of either heterotopic bone or vascular compromise [7,8].

To position the present case within the contemporary published experience, we draw on three complementary streams of recent evidence: the imaging-based AVN surveillance work [15], the 2024 narrative-review synthesis of femoral head reconstruction technique [16], and the foundational early-reduction series that anchors the historical comparator data [11]. The principal series and reviews using the Ganz approach for femoral head fractures are summarised in Table 2 alongside outcomes reported with traditional approaches.

**Table 2:** Comparison of surgical approach for Pipkin type I and II femoral head fracture: contemporary published series. AVN: avascular necrosis; HHS: Harris Hip Score; HO: heterotopic ossification; SHD: surgical hip dislocation; SPECT: single-photon emission computed tomography. AVN rates with traditional anterior or posterior approaches in mixed-type series are typically reported between 6% and 24% [4,5,11,16]. The present case (n=1) is included for narrative positioning only and not as comparative data of equivalent evidence level. The absence of avascular necrosis at 12 months in the present case reflects the current status under continued surveillance, not a definitive outcome, as post-traumatic avascular necrosis of the femoral head may present up to five years following injury.

Study	n	Approach	AVN rate	HO ≥ Brooker II	Functional outcome (Excellent/Good)
Swiontkowski et al. (1992) [5]	24	Anterior (Smith-Petersen) vs. Posterior (Kocher-Langenbeck)	0% anterior; 0% posterior	Higher with anterior approach	Comparable; anterior preferred for visualisation
Marchetti et al. (1996) [4]	33	Mixed (mostly anterior/posterior)	23%	Up to 30%	54%
Massé et al. (2015) [10]	13	Ganz surgical hip dislocation	0%	0%	83%
De Mauro et al. (2021) [12]	9	Gibson approach + Ganz osteotomy	0%	0%	100% satisfactory short-term
Hosny et al. (2022) [14]	18 (12 type II)	Ganz surgical hip dislocation	5.5% (1/18)	Not specified	83% type II excellent/good
Yoon et al. (2022) [15]	34	Surgical hip dislocation with MRI/SPECT surveillance	Low (imaging-confirmed)	Not specified	Favourable midterm
Sarkar et al. (2024) [17]	6	Ganz surgical hip dislocation	16.7% (1/6, late presentation)	0%	83% excellent
Present case (2026)	1	Ganz SHD + headless compression (Herbert) screws	None at 12 months; surveillance ongoing	0%	Excellent (HHS 98)

Three observations emerge from this synthesis. First, all published Ganz-based series report AVN rates at or below the upper bound (5.5% to 16.7%) seen in mixed-approach historical series, with the single outlier (Sarkar et al., 16.7%) attributable to a delayed open reduction following failed closed reduction rather than to the approach itself [17]. Second, the Ganz approach is consistently associated with very low rates of clinically significant heterotopic ossification, in contrast to the Smith-Petersen approach. Third, despite this favourable trend, the published literature remains thin in granular operative detail; the largest series describe outcomes rather than reproducible technique.

The implant choice in this case warrants brief comment. A femoral head fragment of the size encountered here (22 by 18 mm) requires interfragmentary compression to permit early healing, but the implant must also disappear entirely beneath the articular surface against which it will articulate after the head is reduced into the acetabulum. A conventional partially threaded cancellous screw fails the second requirement: its head either rides proud and abrades the acetabular cartilage on every step taken or must be sunk into the cartilage itself, creating an iatrogenic chondral crater. Bioabsorbable implants were attractive in principle a decade ago, but the experimental evidence has been mixed - lower stiffness, osteolysis around incompletely resorbed polymers, and unpredictable absorption times have all been reported [18]. The Herbert-style headless compression screw resolves this competing requirement neatly: the differential pitch between leading and trailing threads generates compression as the screw advances, the entire implant sits within bone once countersunk, and no part of the construct ever contacts the opposing articular surface. Most series of femoral head fixation via the Ganz window have, nonetheless, reported standard 3.5 mm or 4 mm cancellous screws. A previous Cureus case report described this combination in an obese patient but provided limited stepwise technical detail [19].

The 12-month outcome in the present patient (Harris Hip Score 98, modified Merle d’Aubigné-Postel 17, Tönnis 0, Brooker 0) is consistent with the favourable trend seen in the published Ganz series and exceeds the average functional outcome reported with traditional approaches. Continued surveillance remains essential, however, as avascular necrosis can declare itself between 2 and 5 years post-injury [14]; the imaging-based surveillance protocol described by Yoon et al. [15] using sequential MRI and SPECT may be considered for longer-term monitoring of cases at perceived higher risk. A recent systematic review of surgical hip dislocation for femoral head trauma confirmed the favourable AVN profile of this approach but identified persistent gaps in reproducible technical reporting at the case level [20].

Strengths, limitations, and novelty

The principal contribution of this report is not the discovery of a new technique or indication, both of which are now well established in a series of higher levels of evidence [10,12,14,15,16,17]. Rather, this case provides a stepwise, high-fidelity technical description that complements the existing series-level literature. Specific strengths include structured serial outcome assessment using multiple validated scoring instruments (Harris Hip Score, modified Merle d’Aubigné-Postel, Brooker, and Tönnis) rather than a single endpoint; explicit documentation of the time-to-reduction interval (3 hours 40 minutes), which is the dominant modifiable predictor of avascular necrosis [11,15]; and direct contextualisation of the present outcome against contemporary published series in a comparative table. The combination of the Ganz approach with countersunk headless compression screw fixation, while individually well-described, is less frequently reported as a paired technique in the available literature.

This report has several limitations. It describes the experience of a single patient, precluding generalisability and statistical inference. The 12-month outcome, while favourable, does not exclude the possibility of late-onset avascular necrosis or post-traumatic osteoarthritis, both of which can manifest 2 to 5 years after injury; continued long-term follow-up is therefore essential. The case represents the experience of one surgical team in a single institution, and outcomes with the Ganz approach are known to be sensitive to surgeon volume and training [12]. Larger comparative prospective series, ideally with imaging-based AVN surveillance, are needed to definitively position the Ganz surgical hip dislocation against traditional approaches for this indication, and synthesis of such evidence in updated review form will be essential for translating accumulated experience into standardised practice [16]. We hope that the detailed operative narrative provided here will be of educational value to surgeons and trainees adopting this approach.

## Conclusions

The Pipkin type II fragment occupies a difficult middle ground in hip trauma surgery. It is small enough to appear deceptively minor on a radiograph, yet because it carries a portion of the weight-bearing dome, even a millimetre of malreduction translates into kinematic abnormality at the arc of motion the patient will load for the rest of their life. The surgeon’s task is therefore unforgiving on two fronts at once: the fragment must be restored to anatomy without step or gap, and the femoral head’s already-compromised blood supply must be preserved in the process. The Ganz surgical hip dislocation, as practised in this case, addresses both requirements by delivering the femoral head into the wound on its preserved medial circumflex pedicle, while a countersunk headless compression screw construct holds the fragment without ever contacting the opposing acetabular surface.

In our patient, this combination produced a functional outcome indistinguishable from the uninjured side at one year, with no radiographic evidence of avascular necrosis, joint space narrowing, or heterotopic ossification. The intent of this report is not to claim novelty, as the approach, the implant, and their combination have all been previously described, but to provide the granular operative detail that trainees and adopting surgeons need at the table and that series-level reports understandably compress. Late-onset avascular necrosis remains a real possibility at two to five years, and continued surveillance is therefore essential, but the present experience supports the view that the technical sequence is reproducible enough to be taught.
